# Ileocolic resection is associated with increased susceptibility to injury in a murine model of colitis

**DOI:** 10.1371/journal.pone.0184660

**Published:** 2017-09-18

**Authors:** Troy Perry, Michael Laffin, Richard N. Fedorak, Aducio Thiesen, Bryan Dicken, Karen L. Madsen

**Affiliations:** 1 Department of Surgery, University of Alberta, Edmonton, Alberta, Canada; 2 Department of Medicine, University of Alberta, Edmonton, Alberta, Canada; 3 The Centre of Excellence for Gastrointestinal Inflammation and Immunity Research (CEGIIR), University of Alberta, Edmonton, Alberta, Canada; 4 Department of Laboratory Medicine, University of Alberta, Edmonton, Alberta, Canada; Cincinnati Children's Hospital Medical Center, UNITED STATES

## Abstract

Ileocolic resection (ICR) is the most common intestinal resection performed for Crohn's disease, with recurrences commonly occurring at the site of the anastomosis. This study used an animal model of ICR in wild-type mice to examine immunologic changes that developed around the surgical anastomosis and how these changes impacted gut responses to minor acute injury. ICR was performed in adult 129S1/SvlmJ mice and results compared with mice receiving sham or no surgery. Dextran sodium sulfate was given either on post-operative day 9 or day 24 to evaluate immune responses in the intestine both immediately following surgery and after a period of healing. Fecal occult blood measurements and animal weights were taken daily. Cytokine levels were measured in ileal and colonic tissue. Bacterial load in the neo-terminal ileum was measured using qPCR. Immune cell populations in the intestinal tissue, mesenteric lymph nodes, and spleen were assessed using flow cytometry. Cytokine secretion in response to microbial products was measured in isolated mesenteric lymph nodes and spleen cells. ICR resulted in an initial elevation of inflammatory markers in the terminal ileum and colon followed by enhanced levels of bacterial growth in the neo-terminal ileum. Intestinal surgical resection resulted in the recruitment of innate immune cells into the colon that exhibited a non-responsiveness to microbial stimuli. DSS colitis phenotype was more severe in the ileocolic resection groups and this was associated with local and systemic immunosuppression as evidenced by a reduced cytokine responses to microbial stimuli. This study reveals the development of an immune non-responsiveness to microbial products following ileocolic resection that is associated with enhanced levels of bacterial growth in the neo-terminal ileum. These surgical-induced altered immune-microbial interactions in the intestine may contribute to disease recurrence at the surgical anastomosis site following ileocolic resections in patients with Crohn’s disease.

## Introduction

Surgical resection of the intestine is required for the majority of Crohn’s disease (CD) patients at some point in the course of their disease [1]. Owing to disease location, resection of the ileocolic (ICR) region is the most common intestinal resection performed. The natural history of disease recurrence following ICR is well-described with rapid and uniform recurrence in the neo-terminal ileum [2]. Investigations focusing on microbiology and post-operative recurrence have found that ICR leads to an increase in total bacteria in the neo-terminal ileum and that CD patients with recurrence experience an associated microbial dysbiosis [3–5]. These observations highlight the association of microbes with recurrence, but little is known about the immunologic changes that accompany ICR or why disease tends to occur at the site of the anastomosis. Mononuclear phagocytes, including macrophages and dendritic cells (DCs), are found in the gut lamina propria where they link innate and adaptive response to gut antigens. Homeostasis under normal conditions is maintained by lamina propria macrophages through phagocytosis of microbes with minimal inflammatory potential [6] while DCs promote tolerance by inducing T regulatory cells (Tregs) [7]. Both phagocyte cell types demonstrate functional shifts with intestinal inflammation as well as in response to injury [8–11].

The post-operative period is commonly thought to consist of a combination of inflammation and healing. However, there is a growing body of literature on the emergence of immunosuppression as a consequence of surgery [12, 13]. Post-operative systemic immunosuppression is thought to be mediated by a variety of factors collectively attributed to ‘surgical stress’ [14]. These include changes in neuroendocrine, paracrine, and hormonal secretions, along with changes in the behavior of specific immune cell populations [12, 15]. Although bowel resection is a commonly performed procedure, knowledge concerning the resultant local immunologic response is not well described. Experiments performed in the IL-10^-/-^ mouse model of ICR demonstrated that following surgery, significant changes in microbiota in the neo-terminal ileum are seen along with a post-operative ileitis (16–18). This study used an animal model of ICR in wild-type mice to examine immunologic changes occurring around the surgical anastomosis and to determine how these changes impact gut responses to minor acute injury. The goal was to provide insight into local immune and microbial changes induced by ICR that may interact together to predispose disease recurrence at the site of the anastomosis.

## Materials and methods

### Animal model

Animal use protocols were approved by the Health Science Animal Care Committee at the University of Alberta. Wild type 129S1/SvlmJ mice (12–13 weeks old) underwent ICR with primary end-to-end anastomosis as previously described [16]. Animals were put on a liquid diet (LD101 test diet) for 24 hours prior to surgery. The ileum was then transected 2 cm proximal to the ileocecal junction and the ascending colon divided just distal to the cecum. The ileocolic anastomosis was constructed with 8–0 Prolene (Ethicon US, LL)., Cincinnati, OH on a tapered needle. The abdominal wall was then closed with a running 5–0 silk suture and isoflourane was discontinued. Animals recovered under a heat lamp until alert after which they were placed into a clean cage and maintained on the liquid diet for 2 days. To control for the effects of surgical stress, mice in the sham surgery group (sham) underwent a sham procedure in which the ileocecal region was expressed from the abdomen via a midline incision, exposed to air for 10 minutes, replaced, and the laparotomy was closed. Overall survival in the operative groups was 97%. Animals in the non-operative control group (Ct) were also fed a liquid diet for 3 days.

### Animal experimental procedures post-ICR

**Fig 1:**
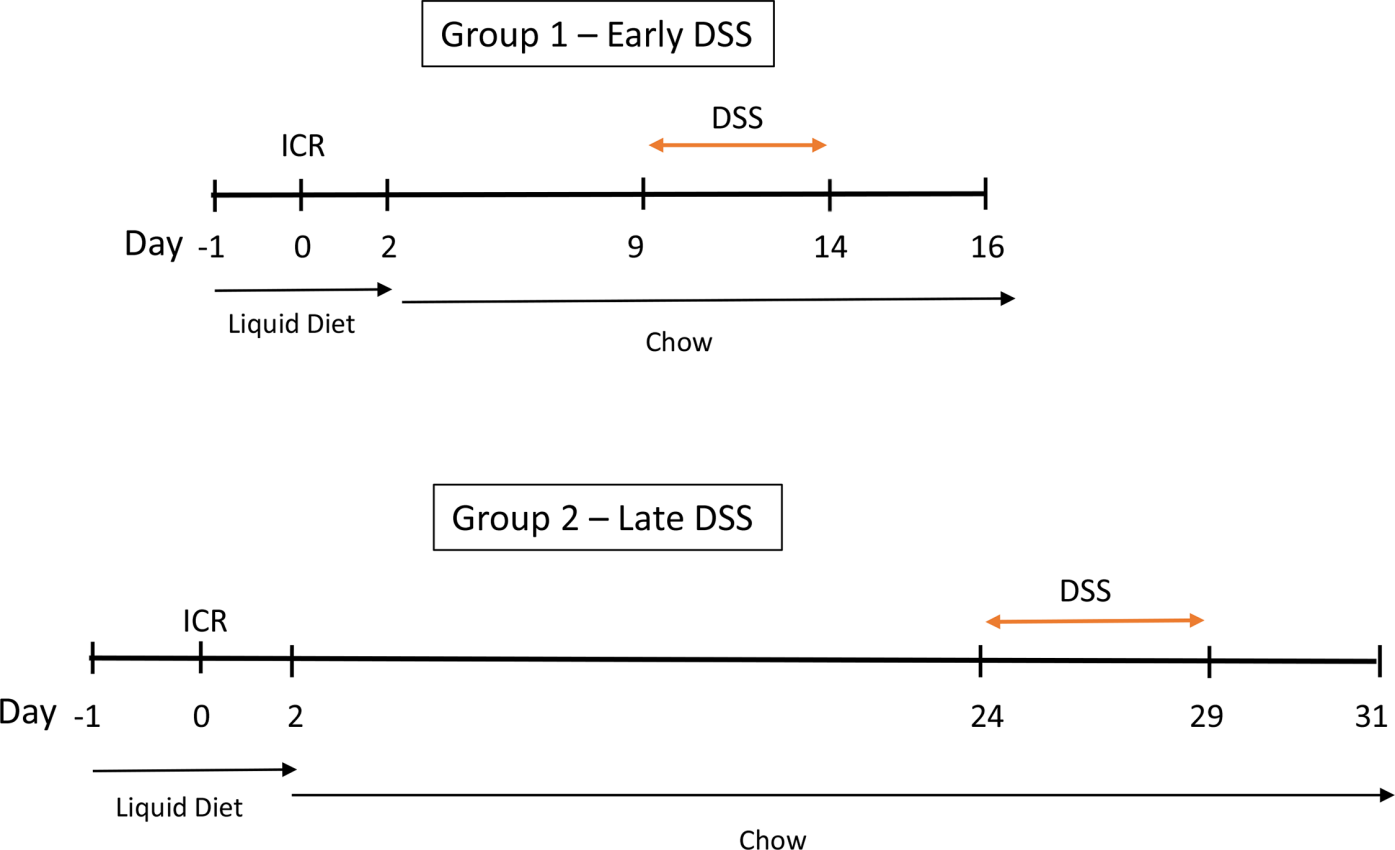
Timeline of experiments in two separate cohorts of ICR mice. All mice were placed on a liquid diet one day prior to surgery. Following ICR, mice remained on a liquid diet for 2 days, and then were placed back on chow diet. In Group 1, DSS was administered between days 9 and 13 post-op, followed by water for 2 days. Mice were sacrificed on Day 16 and tissues removed for analysis. In Group 2, DSS was administered between days 24 and 28 post-op, followed by water for 2 days. Mice were then scarified on day 31 and tissues removed for analysis.

Following ICR, mice were treated with a low concentration of dextran sodium sulfate (DSS 2.5%; MW 35–45,000 kDa; MP Biomedicals). Two time points following ICR were studied to determine the impact of a minor acute insult occurring either early (9 days post-operatively: Group 1 –Early DSS) or after a longer period of healing (24 days post-operative: Group 2 –Late DSS). A timeline is shown in Fig 1. Animal groups studied were non-operative controls (Ct) ± DSS, sham surgery (Sh, or operative controls) ± DSS, and ICR ± DSS. DSS was added to the drinking water for 5 consecutive days followed by a recovery period of two days. Mice were euthanized on day 16 (early DSS) or day 31 (late DSS) and tissues collected at that time for analysis. Mice were euthanized by inducing deep anesthesia with inhaled Isoflurane followed by cervical dislocation and tissues harvested. Body weight, stool consistency, and fecal occult blood (FOB) were measured every 2 days during the protocol. Animals were monitored using institutional protocols for signs of distress and excessive weight loss and were euthanized if any endpoints were met. FOB positivity was determined using the Hemoccult (Beckman Coulter) test. The weight to length ratio was determined for colon and ileum prior to division for analysis. Because of anticipated effects in the neo-terminal ileum (nTI) following ICR, the intestinal weight to length ratios included 12cm of the distal ileum in addition to the colon.

### Measurement of cytokines and myeloperoxidase

Snap frozen peri-anastomotic colon and ileum were homogenized in PBS containing 0.05% Tween 20. Homogenates were centrifuged at 10 000 rpm for 10 minutes. Cytokine levels were corrected for dry tissue weight. IFN-γ, IL-1β, IL-10, IL-12 p70, IL-2, IL-4, IL-5, IL-6, KC/GRO, and TNF-α were evaluated using the Proinflammatory Panel 1 V-PLEX Mouse Kit (Meso Scale Discovery, Rockville, MD) as per manufacturer’s protocol. IL-10, IL-6, TNF-α, and TGF-β also measured using the ELISA duo sets (R&D Systems, Inc., Minneapolis, MN). Myeloperoxidase (MPO) was measured using the MPO, Mouse, ELISA kit (Hycult Biotech Inc., Plymouth Meeting, PA).

### Immunofluorescence

Tissues for immunofluorescence studies were placed in OCT medium then flash frozen prior to storage at -80°C. Intestinal tissues were cut using a cryostat at 6μm then fixed and permeabilized in acetone at -20°C for 10 minutes. After, tissues were blocked with 10% goat serum for 30 minutes, rinsed in PBS (3x) and stained with primary antibodies in PBS with 0.5% BSA overnight at 4°C. Slides were rinsed again with PBS and secondary antibodies were applied for 1 hour at room temperature, rinsed, and counterstained with 4',6-diamidino-2-phenylindole (DAPI) and FlourSave mounting media was applied (Calbiochem; Millipore Canada Ltd., Etobicoke, ON). Tissues were viewed with a Zeiss Axio fluorescence microscope equipped with using ZEN blue software; cells were quantified as previously described[17].

### 16s rRNA qPCR for tissue bacterial load

Sections of ileum taken 1cm from the anastomosis site were snap frozen. Genomic DNA was extracted using the FastDNA Spin Kit (MP Biomedicals, Santa Ana, CA) following manufacturer’s protocol, and quantified on an ND-1000 NanoDrop spectrophotometer (Thermo Fisher Scientific, Waltham, MA). Samples were diluted to 50ng/ml. Diluted DNA specimens were then quantified again prior to Quantitative PCR (Qpcr) using the PicoGreen assay (Invitrogen, Life Technologies Inc., Burlington, ON). qPCR reactions contained 8μl H_2_O, 10μl of Fast SYBR Green Master Mix, 1μl each of 10μM forward and reverse primers and 2μl target DNA. Sequence of PCR conditions was 5 minutes at 50°C, 5 minutes at 95°C, (15 seconds at 95°C, 1 min at 60°C)x40 cycles, followed by a melting curve step, progressing from 60°C to 95°C, over 12 minutes. qPCR was performed in MicroAmp 96 well optical plates with the 7900HT instrument; results were analyzed with SDS 2.3 software. Non-specific amplification was determined using melting curves and visualizing products on a QIAxcel (Qiagen Inc., Toronto, ON) instrument. All qPCR reagents and materials were obtained from Applied Biosystems unless otherwise stated. Target DNA copy number was determined by comparison to standard curves constructed from purified PCR product obtained using the primers and quantified using the PicoGreen assay (Invitrogen) to amplify genomic DNA from stool in standard PCR reactions [18]. Gene copy number per gram tissue was then determined.

### Isolation and characterization of lamina propria cell and splenocyte populations

Splenocytes and mesenteric lymphocytes from all mice were isolated by individually homogenizing spleens and mesenteric lymph nodes in PBS with 5% FBS, and passing through a 70μm cell strainer. Red blood cells were lysed by osmotic shock. Cells were washed in PBS, passed through a 40μm cells strainer, and aliquoted for analysis after counting. Details of isolation have been described previously [19].

### Characterization of mononuclear phagocytes

Isolated splenocytes and mesenteric lymphocytes were re-suspended in triplicate in PBS and incubated for 30 minutes at 4°C with LIVE/DEAD fixable aqua dead cell stain kit (Invitrogen). Cells were washed in PBS with 5% FBS and incubated for 20 min with antibody cocktail containing PerCP-conjugated anti-mouse CD45, PE-conjugated anti-mouse CD103, APC-conjugated anti-mouse CD11c, AlexaFluor 700-conjugated anti-mouse I-Ab, eFluor 450-conjugated anti-mouse CD11b, and PE-Cy7-conjugated anti-mouse F4/80. Following antibody incubation, cells were fixed in 2% paraformaldehyde in PBS and analyzed on a BD Biosciences FACS Canto II flow cytometer.

### Stimulation of splenocytes and lamina propria cells

Isolated cells (10^6^) from the spleen and lamina propria were pipetted into a 96 well cell culture plate and incubated in cell culture media or cell culture media containing fecal slurry (50mg protein/ml) or cell culture media containing lipopolysaccharide (LPS, 1μg/ml) (Sigma-Aldrich) for 24 hours. Fecal slurries for cell stimulation were created by collecting stool from experimental animals the day prior to sacrifice and homogenizing in PBS, the homogenate was then autoclaved and protein concentration measured with a Bradford assay. Isolated cells from each animal were stimulated with their own stool homogenate to determine response to commensal microbiota. Following incubation, culture media was preserved at -80°C for measurement of secreted cytokines.

### Statistical analyses

All data are expressed as the mean ± SEM. Statistical analysis was performed using Graphpad Prism version 5.04 and mean values compared for significant differences using Student’s one-way analysis of variance with Tukey-Kramer post hoc test. Individual pairwise comparisons were performed with the Mann-Whitney-U test.

## Results

### ICR increases susceptibility to colitis

Following surgery, all ICR mice showed a drop in weight. In the first cohort, DSS was administered to the mice from day 9 to day 13 post-op followed by 2 days of water to examine early immunological changes and responses to acute insults following surgery (Fig 1).

**Fig 2:**
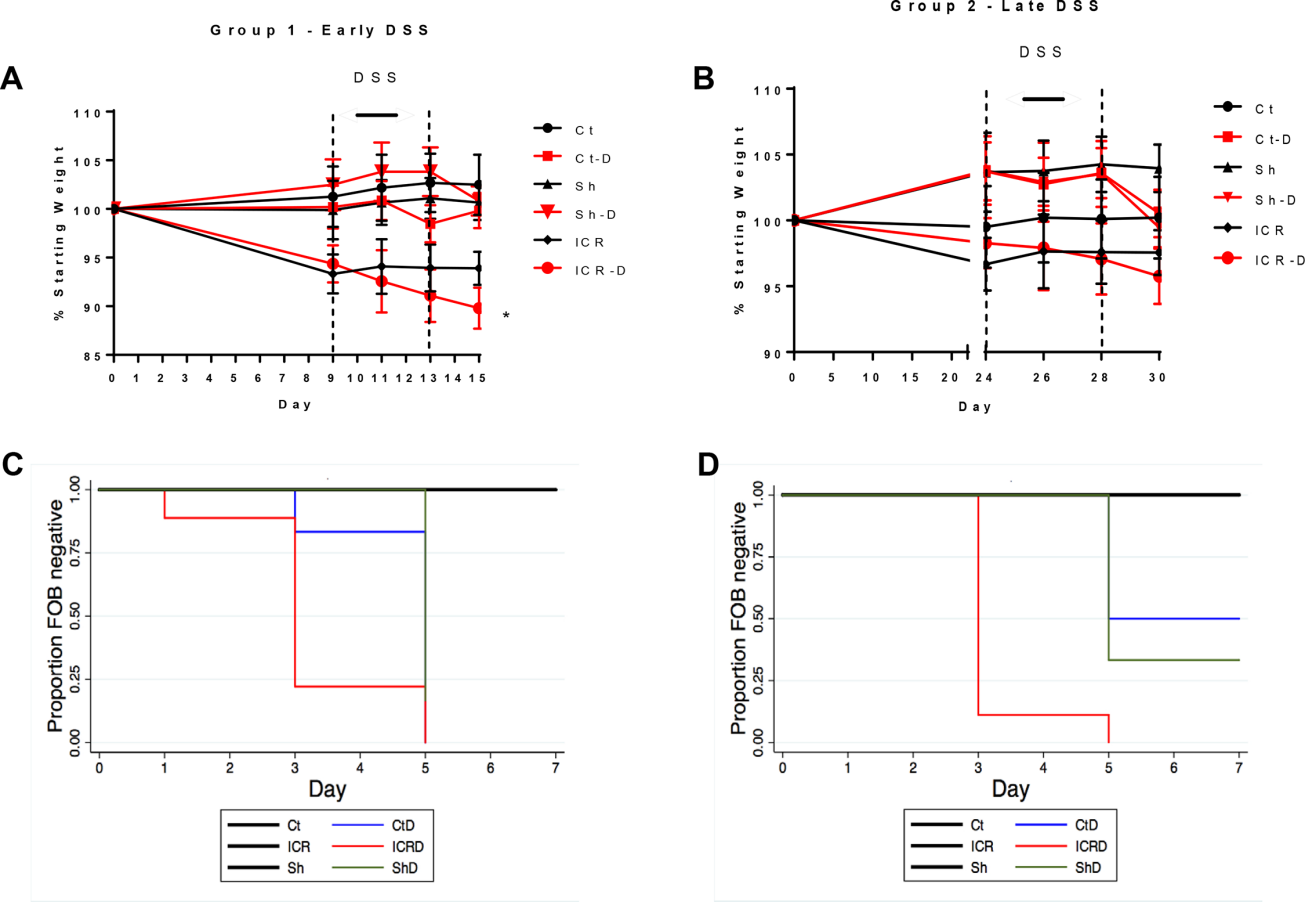
Weight loss and fecal occult blood in stool in response to DSS. n = 6 for Ct and Sh groups; n = 9 for ICR groups. Values are given as Mean ± SEM. Experiments were performed 2x with n = 3 in each of the control and sham groups, and 3x with an n = 3 for the ICR groups. (A) Animal weight from day of surgery (Day 0) to DSS treatment (Day 9–13). Mice in the ICR-D group showed significant weight loss by day 15. *p<0.05 compared with Ct, Ct-D, Sh, and Sh-D. (B) Animal weight from day of surgery (Day 0) to DSS treatment (Day 24–28). There were no significant changes in body weight over the course of the experiment. (C,D) Kaplan-Meier curves demonstrating time to occult blood positive stools. Significance was determined using the log-rank test for equality in survivor analysis. ICR mice showed a significantly earlier onset of occult blood positive stools compared with Ct-D and Sh-D groups in both the early DSS group (C) and in the late DSS group (D).

DSS caused significant weight loss in ICR animals (ICR-D) (p ≤0.05) relative to non-operative control and sham animals (Ct-D) (Fig 2A). Fecal occult blood (FOB) also appeared two days sooner in ICR-D mice compared with controls or sham mice receiving DSS (Fig 2C). There was no fecal occult blood or weight loss seen in mice not treated with DSS. In the second cohort, ICR mice were allowed a longer period of healing prior to the initiation of DSS to examine how immune responses to acute insults changed over time. In this group, DSS was administered from day 24–28 post-op again followed by 2 days of water. Fecal occult blood was also seen in these ICR-D mice two days earlier than in the controls or sham mice which received DSS (Fig 2D) (p>0.001). However, there was no significant weight loss in these ICR mice (Fig 2B).

**Fig 3:**
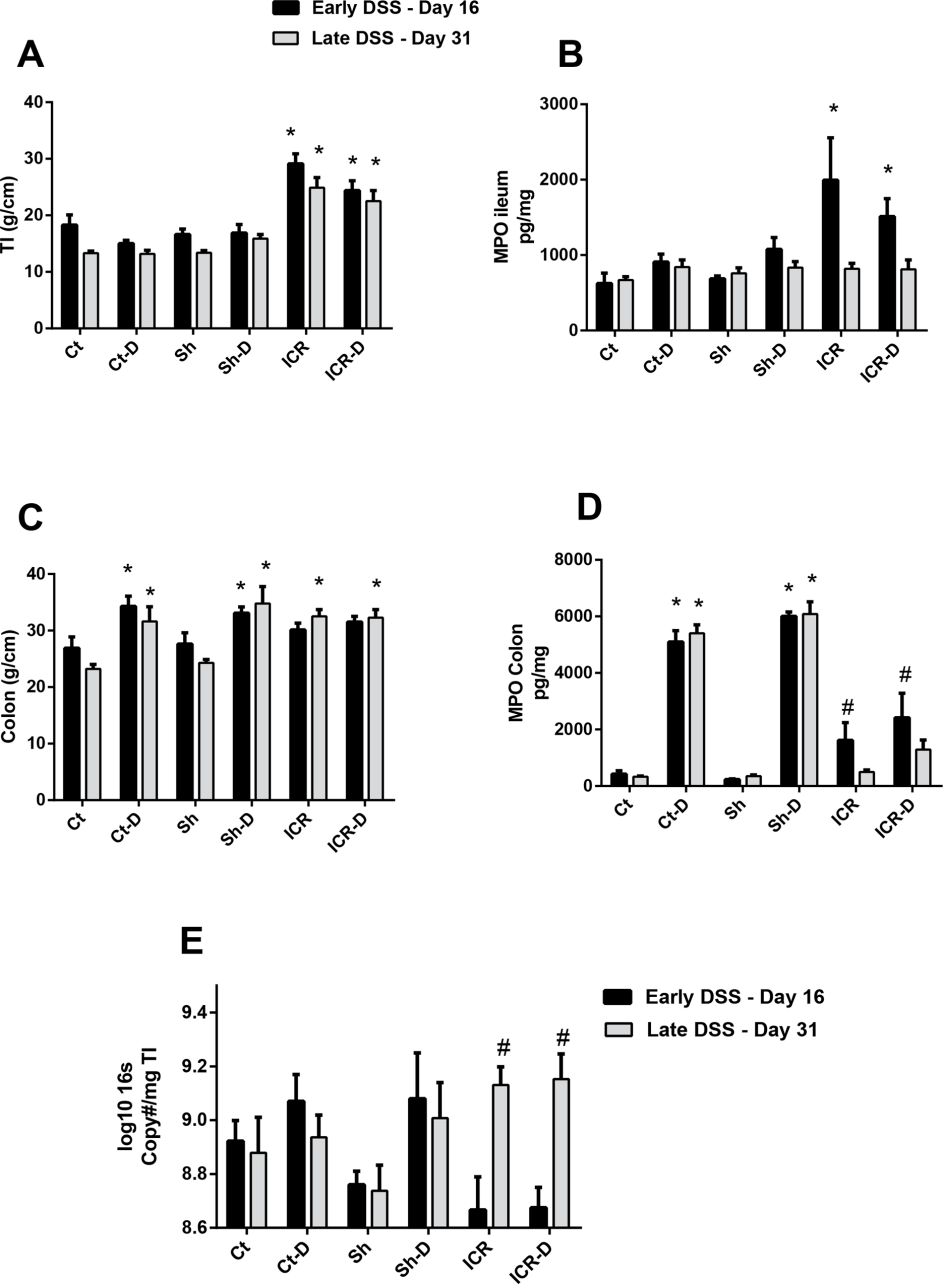
Phenotypic characterization of mice. (A) Tissue weight to length ratios in the ileum. ICR and ICR-D mice had increased weight/length in the neo-terminal ileum at day 16 post-op (early DSS) compared with control and sham mice. This was maintained through day 31 (late DSS). DSS treatment did not induce any further increases in weight/length ratio. *p<0.05compared with Ct, Ct-D, Sh, and Sh-D. (B) MPO levels in ileum. Ileal MPO was increased at day 16 in the ICR and ICR-D mice (Group 1—early DSS) but had returned to control levels by day 31 (Group 2- late DSS). DSS did not induce any changes in MPO levels. *p<0.05 compared with Ct, Ct-D, Sh, and Sh-D. (C) Tissue weight to length ratio in the colon. DSS treatment increased weight/length ratios in control and sham groups. ICR and ICR-D mice had increased weight/length ratios at day 31 post-op (late DSS) compared with controls and sham mice. *p<0.05 compared with Ct and Sh. (D) MPO levels in colon. DSS treatment increased MPO levels in control (Ct-D) and sham (Sh-D) groups. In the ICR mice, MPO was increased at day 16 (Group 1 –early DSS) compared with controls and sham mice. MPO decreased by day 31 (Group 2- late DSS) and was not increased by DSS treatment in the ICR mice. *p<0.01 compared with C and Sh. #p<0.05 compared with Ct and Sh. (E) 16s gene copy number in terminal ileum. There was a significant increase in bacterial load in the ICR groups by day 31 (Group 2 –late DSS). * p≤0.05 relative to Ct and Sh.

Colitis induced by DSS administration is characterized by increased intestinal weight-to-length ratio which correlates with increased inflammation [20]. Tissue samples were taken following DSS treatment at day 16 post-op in the first cohort and at day 31 post-op following DSS treatment in the second cohort. The weight-to-length ratio was increased in the neo-terminal ileum (nTI) of ICR animals relative to controls by day 16 (Group 1;early DSS) and remained increased through day 31 (Group 2;late DSS) (Fig 3A). This increased weight-to-length ratio occurred in response to surgery, as it was seen in the ICR mice in the absence of DSS treatment (p≤0.05 for each comparison).

As expected, there was an increase in colonic weight-to-length ratio in the DSS-treated control and sham groups (Fig 3C). However, in contrast, in the ICR group which was treated with DSS at day 9 post-op, there was no significant increase in colonic weight-to-length ratio when compared with the ICR group. However, this may have been due to the fact that the ICR group already had an increased weight/length ratio, again likely occurring in response to the surgery itself. By day 31, there was an increased colonic weight to length ratio in the ICR group and in the ICR group treated with DSS compared to controls, suggesting a tissue response to surgery had occurred (p≤0.05).

### The neutrophil-mediated response to acute injury is blunted following ICR

Acute insults and resultant wound healing in the intestine are characterized by a recruitment of neutrophils and peripheral blood monocytes to the site of damage. To determine the effect of ICR and DSS injury on neutrophilic infiltration, we measured tissue myeloperoxidase (MPO) levels as a marker. As expected, DSS led to large increases in tissue MPO levels consistent with induction of colitis in the colon of sham and control animals [21]. In contrast, ICR mice did not have a large increase in colonic MPO levels following DSS treatment either at day 16 or at day 31, suggesting that following ICR, there may be an extended period of blunted neutrophil responses to external insults. Interestingly, in the neo-terminal ileum, MPO was elevated at day 16 in both the ICR group and in the ICR group treated with DSS, but by day 31 these levels had returned to levels similar to those seen in control and sham mice.

### ICR increases bacterial load in the neo-terminal ileum

ICR in humans is associated with increased bacterial loads in the terminal ileum, which is thought to occur due to the removal of the ileocecal valve and which contributes to disease recurrence in this region. Therefore, we performed qPCR on genomic DNA extracted from the TI to quantitatively assess total bacterial load (Fig 3E). A trend towards a decreased bacterial load in the ICR and ICR-D groups was seen initially following surgery. This would be expected due to the large influx of oxygen during the surgery itself which would have the effect of killing anaerobes in the area (18). However, by day 31, there was a significant increase in bacterial load in the neo-terminal ileum (p≤0.05).

### ICR blunts the inflammatory response to an acute insult

#### Immune profile in terminal ileum (TI*)*

**Fig 4:**
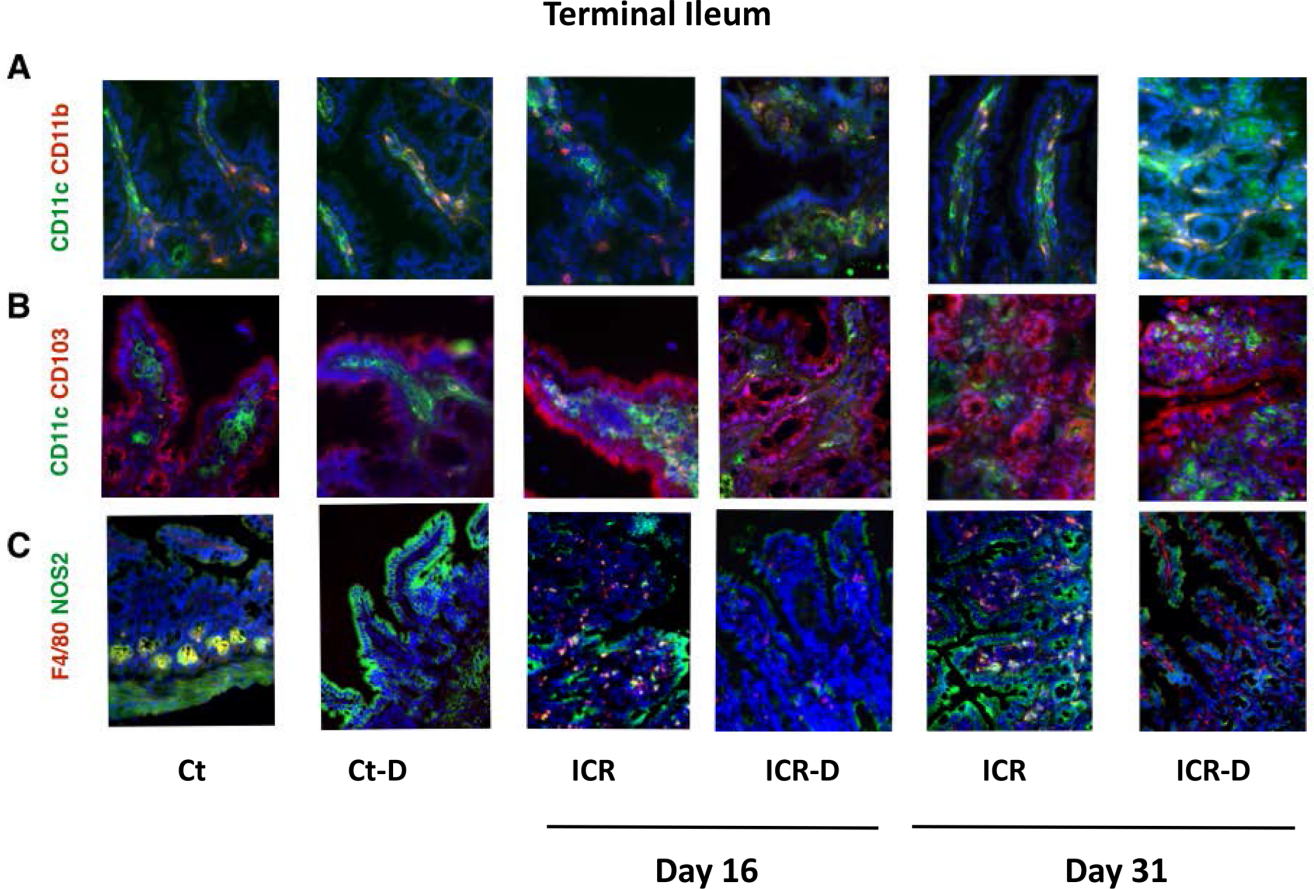
Photomicrographs of double immunofluorescence stained tissue section from colon (40X). (A) Staining for mononuclear phagocyte markers CD11c (Alexa Flour 488-green) and CD11b (Alexa Flour 594-red). CD11c+CD11b- cells were the dominant populations in controls (Ct). DSS and ICR led to a reduction in CD11c+CD11b- cells. CD11c^-^CD11b^+^ cells increased following DSS and by day 31 in ICR group. (B) Staining for migratory dendritic cell populations with CD11c (Alex Flour 488-green) and CD103 (Alexa Flour 594-red). CD11c+CD103+ cells were rarely detected in the villi of non-operative animals but were increased with ICR. This population was typically visualized deep in the lamina propria. (C) Staining for macrophage populations with F4/80 (Alexa Flour 594-red) and NOS2 (Alexa Flour 488-green). F4/80+ cells were dispersed through the lamina propria and increased with DSS and ICR. Co-localization of F4/80 and NOS2 was higher in DSS treated controls and long term ICR groups.

**Fig 5:**
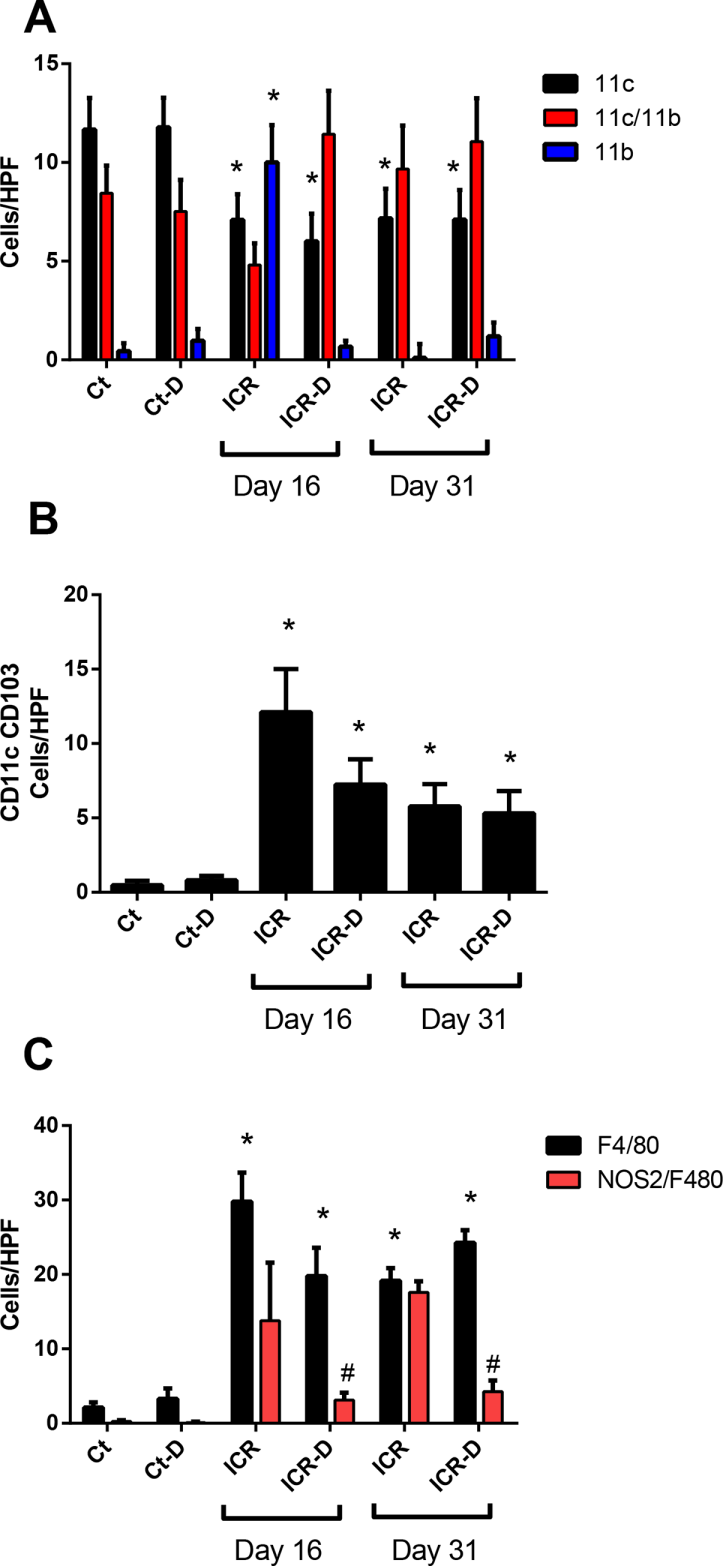
Cell counts from immunofluorescence stained ileal tissue sections. (A) Cells labelled with CD11c and CD11b. At day 16, ICR mice had decreased CD11c+ cells and increased CD11b+ cells. DSS treatment eliminated the rise in CD11b+ cells. At day 31, CD11c+ cells remained decreased in the ICR and ICR-D groups. Data is shown as Mean ± SEM. Counts were performed on 6 representative photomicrographs from n = 3/group. * p ≤ 0.05 relative to Ct. (B) Cells labelled with CD11c and CD103. ICR and ICR-D mice had increased numbers of CD11c+CD103+ cells at day 16 and at day 31. Data is shown as Mean ± SEM. Counts were performed on 6 representative photomicrographs from n = 3/group. * p ≤ 0.05 relative to Ct and Ct-D. (C) Cells labelled with F4/80 and NOS2/F4/80. At day 16 and 31, ICR and ICR-D mice had increased levels of F4/80+ cells. Data is shown as Mean ± SEM. Counts were performed on 6 representative photomicrographs from n = 3/group. * p ≤ 0.05 relative to Ct and Ct-D; # p ≤ 0.05 with DSS.

**Fig 6:**
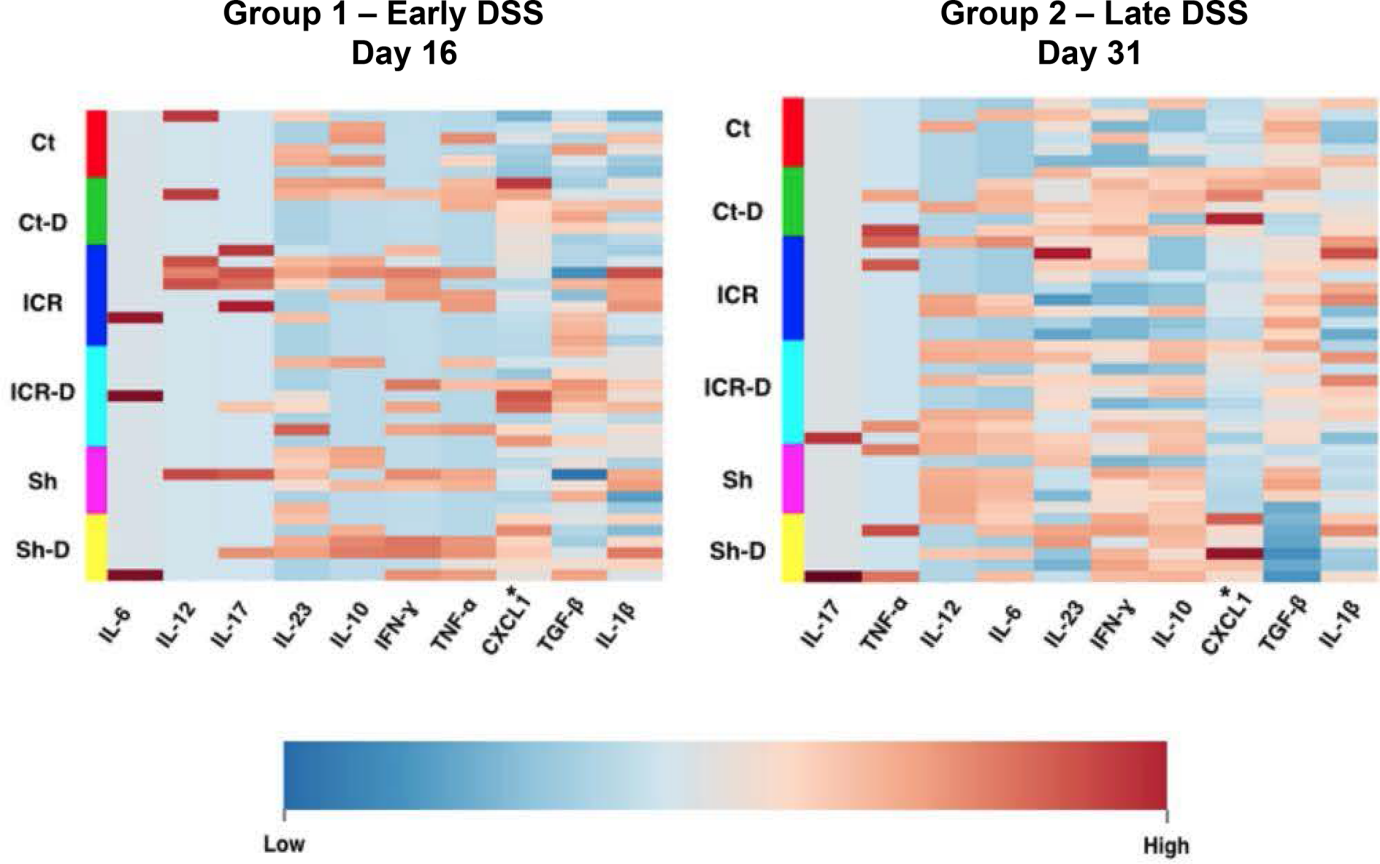
Terminal ileum cytokine profiles did not change after ICR with DSS treatment. Tissue cytokine concentrations were determined using the pro-inflammatory multiplex array (Meso Scale Discovery). Heat Maps were constructed using MetaboAnalyst 3.0. *indicates significantly different across all groups P ≤ 0.005 determined by ANOVA with log transformation. Cytokines in plots are presented in order importance for separation of the groups.

Surgery and DSS would be expected to alter lamina propria innate immune cell populations resulting in both acute and chronic changes in cell populations. Therefore, we performed a characterization of lamina propria mononuclear phagocyte populations in both the neo-terminal ileum and colon at two time points following ICR. Murine mononuclear phagocyte populations are defined by expression of CD11c and CD11b [8, 22]. In the TI of control mice, CD11c^+^ cells represented the dominant population in the lamina propria followed by CD11c^+^CD11b^+^ cells with relatively few single positive CD11b^+^ cells (Figs 4 and 5). DSS alone did not elicit any changes in these ratios in the terminal ileum which was expected in the control mice as DSS induces a colonic injury (Fig 5A). In contrast, significant changes were observed in the neo-terminal ileum of ICR mice. Following ICR there were significant shifts in mononuclear phagocyte subsets with CD11b^+^ cells comprising the majority and a reduction in CD11c^+^ cells (Fig 5A). This would be expected following surgery as CD11b^+^ regulates leukocyte adhesion and migration to modulate inflammatory responses. This increase in CD11b^+^ cells, along with the observed increase in F4/80+ cells, supports the concept of a surgical-induced recruitment of peripheral monocytes to the site of injury in the neo-terminal ileum (Fig 5C). In addition, there was a recruitment of CD11c^+^CD103^+^ cells (Fig 5B) which defines a migratory population of DCs [23]. DSS treatment in the ICR animals led to a further shift with CD11c^+^CD11b^+^ cells becoming the majority. Co-localization of NOS2 with F4/80^+^, which typically labels classical M1 macrophages, was increased with ICR but subsequently decreased with DSS treatment by day 31. CXCL1, primarily defined as a neutrophil chemoattractant was the only cytokine different across the groups in the TI, with elevations in all DSS treated groups except the ICR cohort at day 31 (p≤0.05) (Fig 6).

#### Immune profile in colon

**Fig 7:**
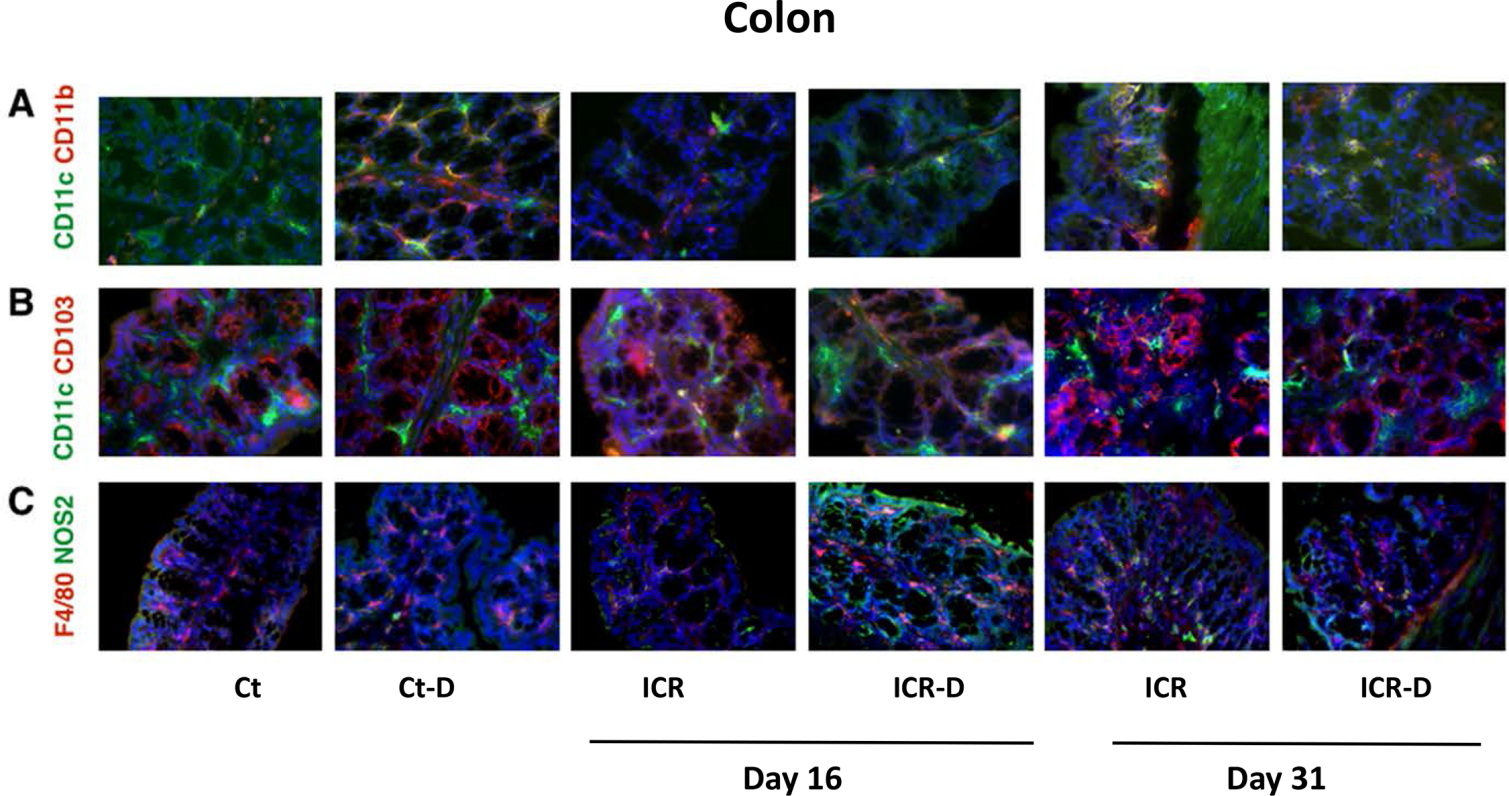
Photomicrographs of double immunofluorescence stained tissue section from colon (40X). (A) Staining for mononuclear phagocyte markers CD11c (Alexa Flour 488-green) and CD11b (Alexa Flour 594-red). CD11c+CD11b- cells were the dominant populations in controls (Ct). DSS and ICR led to a reduction in CD11c+CD11b- cells. CD11c^-^CD11b^+^ cells increased following DSS and/or long term ICR. (B) Staining for migratory dendritic cell populations with CD11c (Alex Flour 488-green) and CD103 (Alexa Flour 594-red). CD11c+CD103+ cells were rarely detected in the villi of non-operative animals but were increased with ICR. This population was typically visualized deep in the lamina propria. (C) Staining for macrophage populations with F4/80 (Alexa Flour 594-red) and NOS2 (Alexa Flour 488-green). F4/80+ cells were dispersed through the lamina propria and increased with DSS and ICR. Co-localization of F4/80 and NOS2 was higher in DSS treated controls and long term ICR groups.

**Fig 8:**
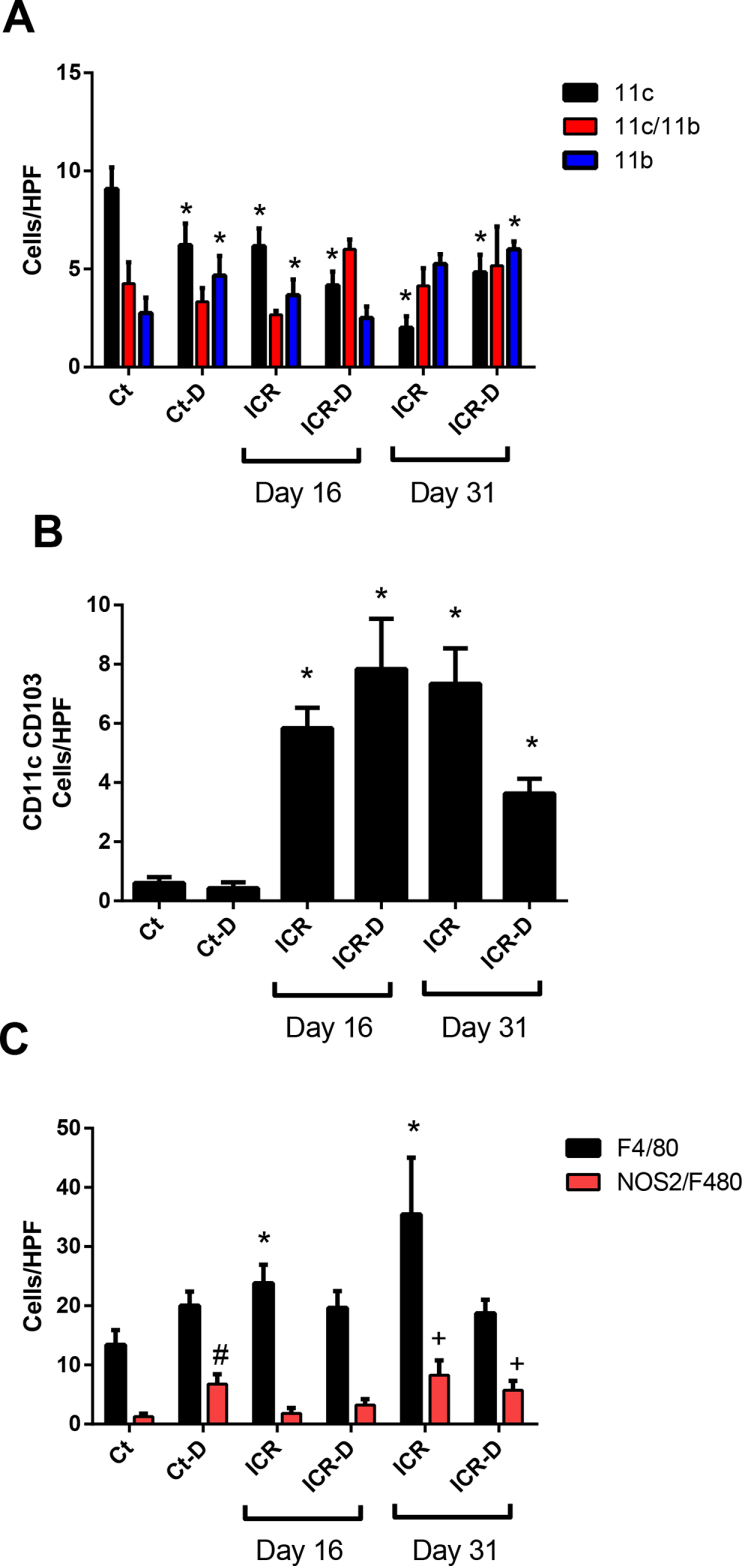
Cell counts from immunofluorescence stained colonic tissue sections. (A) Cells labelled with CD11c and CD11b. In controls, DSS treatment decreased numbers of CD11c+ cells and increased numbers of CD11b+ cells. At day 16, ICR mice had decreased CD11c+ cells and increased CD11b+ cells. DSS treatment in ICR mice eliminated the rise in CD11b+ cells. At day 31, CD11c+ cells remained decreased in the ICR and ICR-D groups, but CD11b+ cells had increased in both groups. Data is shown as Mean ± SEM. Counts were performed on 6 representative photomicrographs from n = 3/group. * p ≤ 0.05 relative to Ct. (B) Cells labelled with CD11c and CD103. ICR and ICR-D mice had increased numbers of CD11c+CD103+ cells at day 16 and at day 31. Data is shown as Mean ± SEM. Counts were performed on 6 representative photomicrographs from n = 3/group. * p ≤ 0.05 relative to Ct and Ct-D. (C) Cells labelled with F4/80 and NOS2/F4/80. At day 16 and 31, ICR and ICR-D mice had increased levels of F4/80+ cells. Data is shown as Mean ± SEM. Counts were performed on 6 representative photomicrographs from n = 3/group. * p ≤ 0.05 relative to Ct and Ct-D; # p ≤ 0.05 with Ct; +p<0.05 compared with Day 16 ICR and ICR-D.

Colonic mononuclear phagocyte subsets were subject to shifts following ICR and in response to DSS treatment. DSS in control mice led to an increase in CD11b^+^ cells with a decrease in CD11c^+^ cells (Figs 7 and 8). ICR alone led to a similar profile, with an increase of CD11b^+^ cells, and decrease of CD11c^+^ cells. However, in contrast to controls, DSS treatment in the ICR group produced a shift toward CD11c^+^CD11b^+^ cells by day 16 but no increase in CD11b^+^. By 31 day in the ICR and ICR-D groups, CD11c^+^CD11b^+^ and CD11b^+^ cells made up the majority, and a further DSS-induced decrease in CD11c^+^ cells was seen. It is likely that many of the CD11c^+^CD11b^+^ cells were DCs as CD103^+^ DCs were elevated across all the ICR and ICR-D groups. Macrophages were also increased following ICR by day 31 and demonstrated increased co-localization of NOS2 suggesting an increase in M1 macrophages (Figs 7 and 8). In the colon, control and sham mice responded to DSS with increased levels of IL-1β, IL-6, IL-23, IL-17, TNFα, IFNγ, and CXCL1 at both day 16 and 31. In contrast, ICR mice did not respond to DSS with increased colonic cytokines at either day 16 (Table 1) or 31 (Table 2).

**Table 1:** Cytokine levels in colonic tissue at day 16.

Condition	Group	Cytokine							
		IL-1β (ng/mg)	IL-6 (ng/mg)	IL-23 (pg/mg)	IL-17 (ng/mg)	TNFα (ng/mg)	IFNγ (ng/mg)	TGFβ (ng/mg)	CXC1 (ng/mg)
**Basal**	Control (n = 6)	1.5 ± 0.1^a^	0.3 ± 0.3^a^	30 ± 19^a^	0.01 ± 0.01^a^	nd	0.01 ± 0.01^a^	nd	3.7 ± 0.6^a^
	Sham (n = 6)	1.6 ± 0.1^a^	nd	62 ± 6^a^	nd	nd	nd	0.1 ± 0.1^b^	6.4 ± 0.4^a^
	ICR (n = 9)	6.7 ± 1.6^a^	nd	59 ± 11^a^	0.09 ± 0.03^a^	0.3 ± 0.1^a^	0.2 ± 0.8^b^	nd	14.1 ± 3.4^a^
**DSS**	Control (n = 6)	143.6 ± 38.4^b^*	32.4 ± 4.6^b^*	130 ± 10^b^*	0.3 ± 0.01^b^*	6.8 ± 1.2^b^*	4.1 ± 0.4^c^*	1.9 ± 0.8^a^*	49.6 ± 10.8^b^*
	Sham (n = 6)	219.2 ± 64.9^b^*	40.9 ± 6.2^b^*	143 ± 24^b^*	0.4 ± 0.08^b^*	8.1 ± 2.2^b^*	5.8 ± 1.7^c^*	2.1 ± 0.8^a^*	49.3 ± 8.5^b^*
	ICR (n = 9)	31.8 ± 14.4^a^	1.9 ± 1.0^a^	70 ± 11^a^	0.04 ± 0.02^a^	0.5 ± 0.2^a^	0.3 ± 0.2^a^	0.2 ± 0.1^b^	26.5 ± 6.3^a^

Values are means ± SEMs.

Labeled means obtained for the same cytokine without a common letter (a,b,c) differ, p<0.05.

*p<0.05 Ct compared with Ct-D; Sh compared with Sh-D

nd: non-detectable

**Table 2:** Cytokine levels in colonic tissue at day 31.

Condition	Group	Cytokine							
		IL-1β (ng/mg)	IL-6 (ng/mg)	IL-23 (pg/mg)	IL-17 (ng/mg)	TNFα (ng/mg)	IFNγ (ng/mg)	TGFβ (ng/mg)	CXC1 (ng/mg)
**Basal**	Control (n = 6)	2.3 ± 0.2^a^	0.01 ± 0.01	25 ± 5^a^	nd	nd	0.02 ± 0.01^a^	8.2±1.4^a^	5.1± 0.4^a^
	Sham (n = 6)	2.3 ± 0.2^a^	0.1 ± 0.01	28 ± 8^a^	0.01 ± 0.01^a^	nd	0.03 ± 0.01^a^	12.8±2.2^a^	4.4 ± 0.3^a^
	ICR (n = 9)	5.3± 1.0^a^	0.05 ± 0.05	26 ± 6^a^	0.01 ± 0.01^a^	nd	0.05 ± 0.02^a^	5.0±0.5^a^	9.1 ± 1.2^a^
**DSS**	Control (n = 6)	286.9 ± 137.3^b^*	58.1 ± 22.0 ^b^*	202 ± 91^b^*	0.7± 0.3^b^*	9.6 ± 4.7 ^b^*	7.9 ± 2.1 ^b^*	6.1 ± 1.1^a^	113.3 ± 47.4^b^*
	Sh-D (n = 6)	275.6 ±117.1^b^*	74.1 ± 25.9 ^b^*	148 ± 35^b^*	1.1 ± 0.4^b^*	12.9 ± 5.4^b^*	12.2 ± 4.7^b^*	5.4± 1.4^a^	86.1 ± 33.7^b^*
	ICR-D (n = 9)	8.4 ±1.4^a^	1.9 ± 1.0^a^	17 ± 5^a^	nd	0.1± 0.1^a^	0.07 ± 0.01^a^	6.0 ± 0.5^a^	13.6 ± 2.1 ^a^

Values are means ± SEMs.

Labeled means obtained for the same cytokine without a common letter (a,b) differ, p<0.05.

*p<0.05 Ct compared with Ct-D; Sh compared with Sh-D

nd: non-detectable

#### Characterization of effects of ICR and DSS on mesenteric lymph nodes

**Fig 9:**
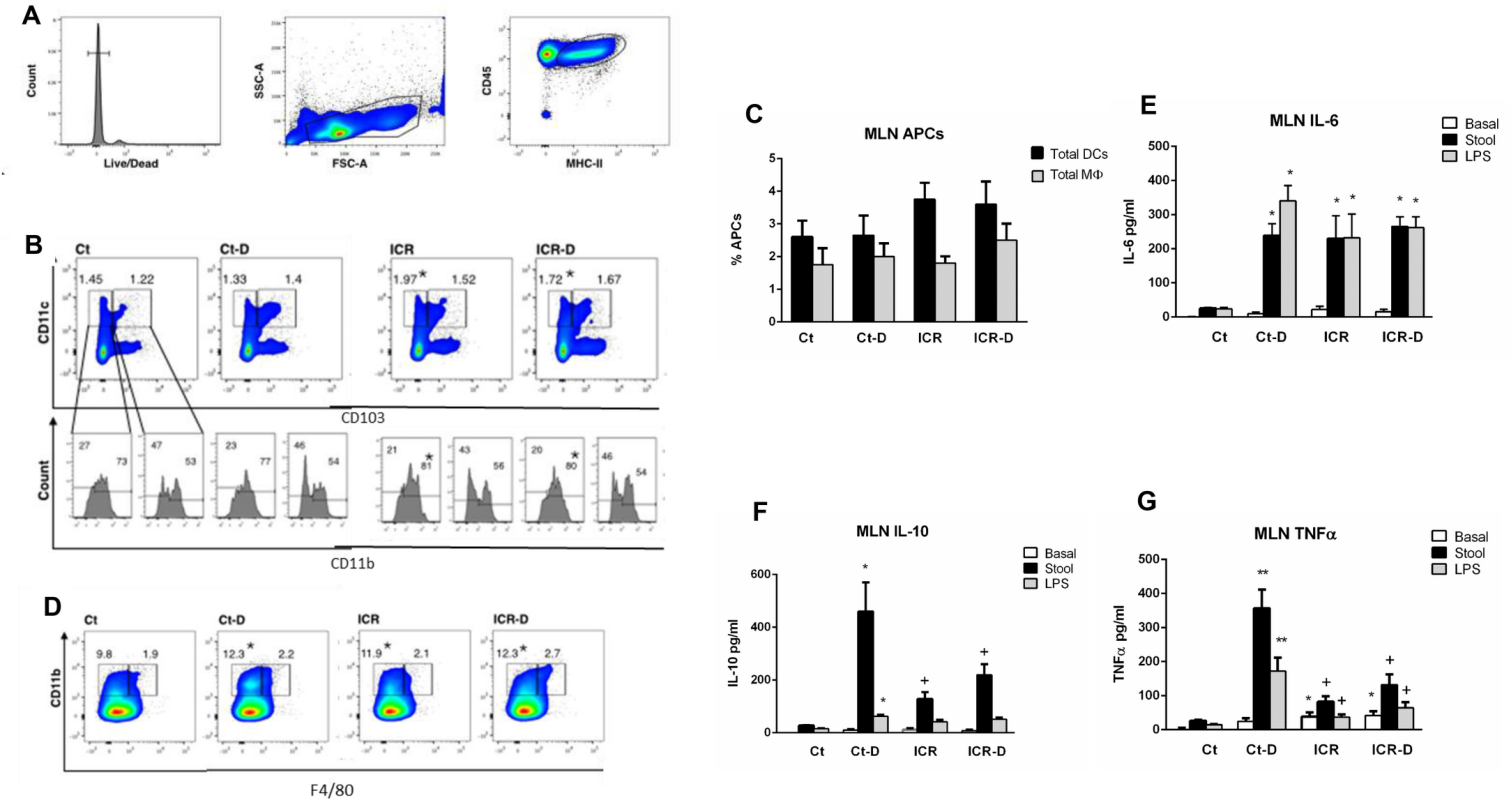
ICR shifts mononuclear phagocyte populations and reduces the inflammatory priming effects of DSS in the mesenteric lymph nodes. (A) Representative gating strategy for identification of antigen presenting cells in the mesenteric lymph nodes. (B) Dendritic cell populations pre-gated as F4/80^-^ antigen presenting cell. Dendritic cell subsets were further defined as based in CD103 and CD11b expression. The CD11c^+^CD103^-^CD11b^+^ subset increased with ICR. (C) Proportion of antigen presenting cells for gated macrophages and dendritic cells in MLN. Values are given as Means ± SEM. (D) Macrophage populations defined as CD11b^+^F4/80^+^ antigen presenting cells. (E) IL-6 secretion from MLN cell cultures under basal conditions and following stimulation with stool slurry (Stool) or LPS, (F) IL-10, and (G) TNFα. Results are representative of 6 independent experiments. * p ≤ 0.05 relative to Ct, + p ≤ 0.05 relative to Ct-D.

Shifts in mononuclear phagocyte populations in the mesenteric lymph nodes (MLNs) in the day 31 mice were evaluated by flow cytometry and functional changes examined with cell culture and quantification of cytokine secretion. To define the mononuclear phagocyte subsets, cells were first gated to identify antigen presenting cells (Fig 9A). Proportions of DC subsets were determined by pre-gating for F4/80^-^ cells to exclude macrophages, followed by quantification of cells as either CD11c^+^CD103^-^ or CD11c^+^CD103^+^. These subsets were further defined by expression of CD11b. In the MLN there was an increase in CD11c^+^CD103^-^ DCs following ICR and an increase in CD11b^+^ expressing cells within this subset. This change persisted following DSS treatment. Total DCs in the MLNs were also quantified by defining cells as CD45^+^MHC-II^+^CD11c^+^F4/80^-^. There was no significant difference across the groups (Fig 9C). Macrophages were quantified as CD45^+^MHC-II^+^CD11b^+^F4/80^+^ cells (Fig 9D). An additional subset of CD11b^+^F4/80^-^ cells was increased in the Ct-D, ICR, and ICR-D groups (Fig 9D).

Stimulation of lymph node isolates with either fecal slurries (stool) or LPS in primary cell culture was performed to determine the functional effects of ICR-induced changes in cellular phenotypes. ICR mice showed a small increase in basal TNFα secretion compared with controls (Fig 9G). MLNs obtained from Ct-D mice showed a large TNFα (Fig 9G), IL-10 (Fig 9F), and IL-6 (Fig 9E) response when stimulated with either stool samples or LPS. In contrast, the ICR mice displayed significantly attenuated TNFα and IL-10 responses to stool and LPS, although IL-6 secretion was similar.

### Characterization of effects of ICR and DSS on systemic immune profile

**Fig 10:**
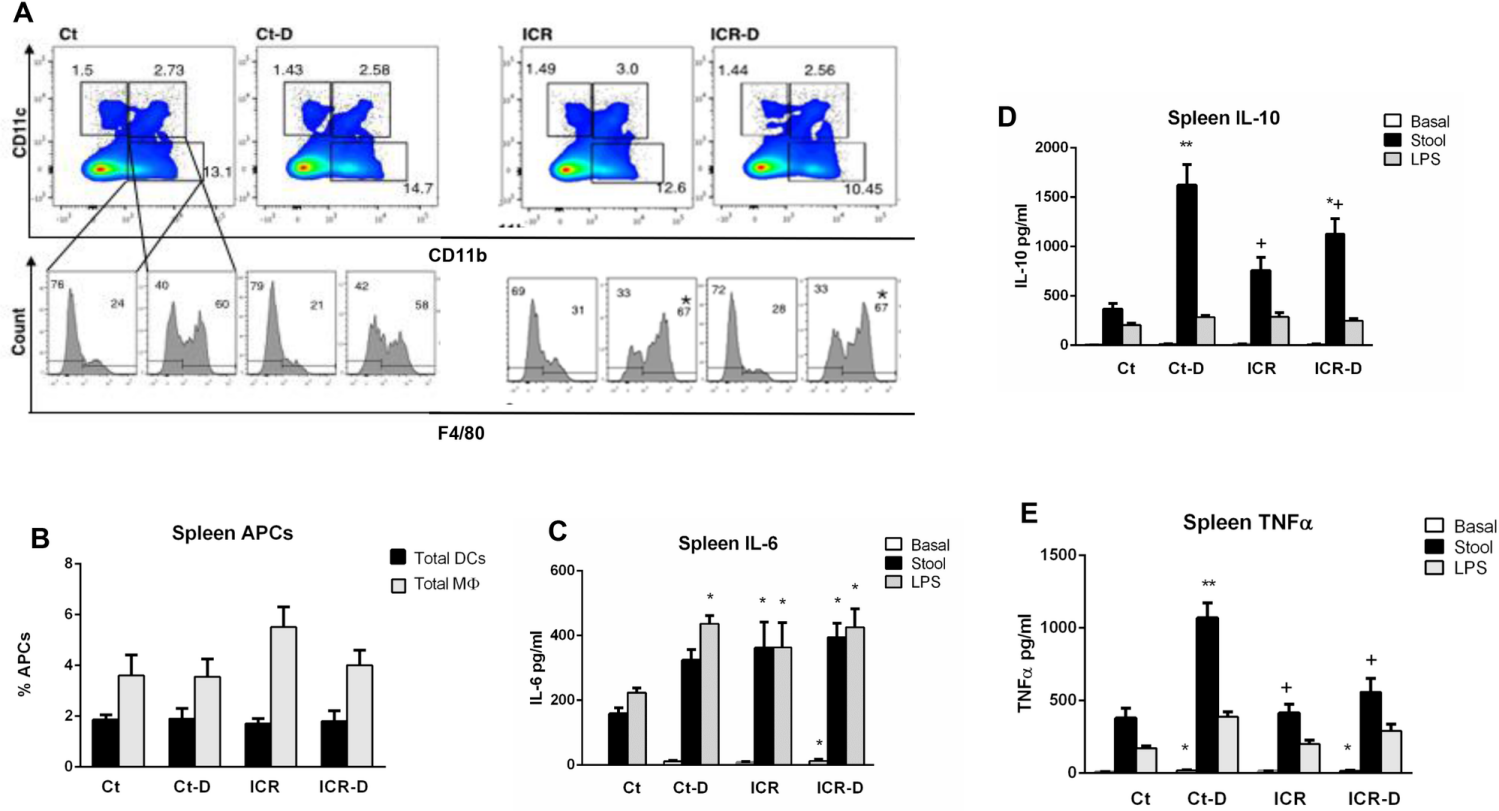
ICR blunts systemic responses. (A) Macrophage and dendritic cell subsets determined by pre-gating for antigen presenting cells followed by identification of CD11c, CD11b and F4/80 expression. (B) Total mean ± SEM proportion of antigen presenting cells for gated macrophages and dendritic cells. (C) IL-6, (D) IL-10, and (E) TNFα secretion from splenic cell cultures under basal conditions and following stimulation with stool slurry (Stool) or LPS. Results are representative of 6 independent experiments. * p ≤ 0.05 relative to Ct, + p ≤ 0.05 relative to Ct-D.

The effect of ICR and DSS on systemic immune profile in the day 31 mice was determined by phenotypic and functional characterization of splenocytes and the measurement of serum cytokines. Flow cytometry demonstrated no significant changes in splenic proportions of CD11c^+^, CD11c^+^CD11b^+^, and CD11b^+^ subsets with ICR or DSS (Fig 10A). Relative proportions of macrophages within the CD11c^+^CD11b^+^ and CD11b^+^ groups were determined by F4/80 expression. ICR caused an increase in F4/80^+^ cells within the CD11c^+^CD11b^+^ subset. When total macrophages and DCs were measured, there was no difference across groups (Fig 10B).

Splenocytes from all groups responded to both stool and LPS with secretion of similar levels of IL-6 (Fig 10C). In the control group, isolated splenocytes responded to stool samples with a large increase in IL-10 and a smaller response to LPS (Fig 10D). The IL-10 response was increased in the ICR group compared with controls, but attenuated in the ICR-D group compared to the control-DSS group. A similar profile was seen for TNFα secretion (Fig 10E). These data suggest that although all groups had similar numbers of DCs and macrophages, functional responses to stool antigens and LPS were induced systemically by intestinal surgery and DSS.

**Fig 11:**
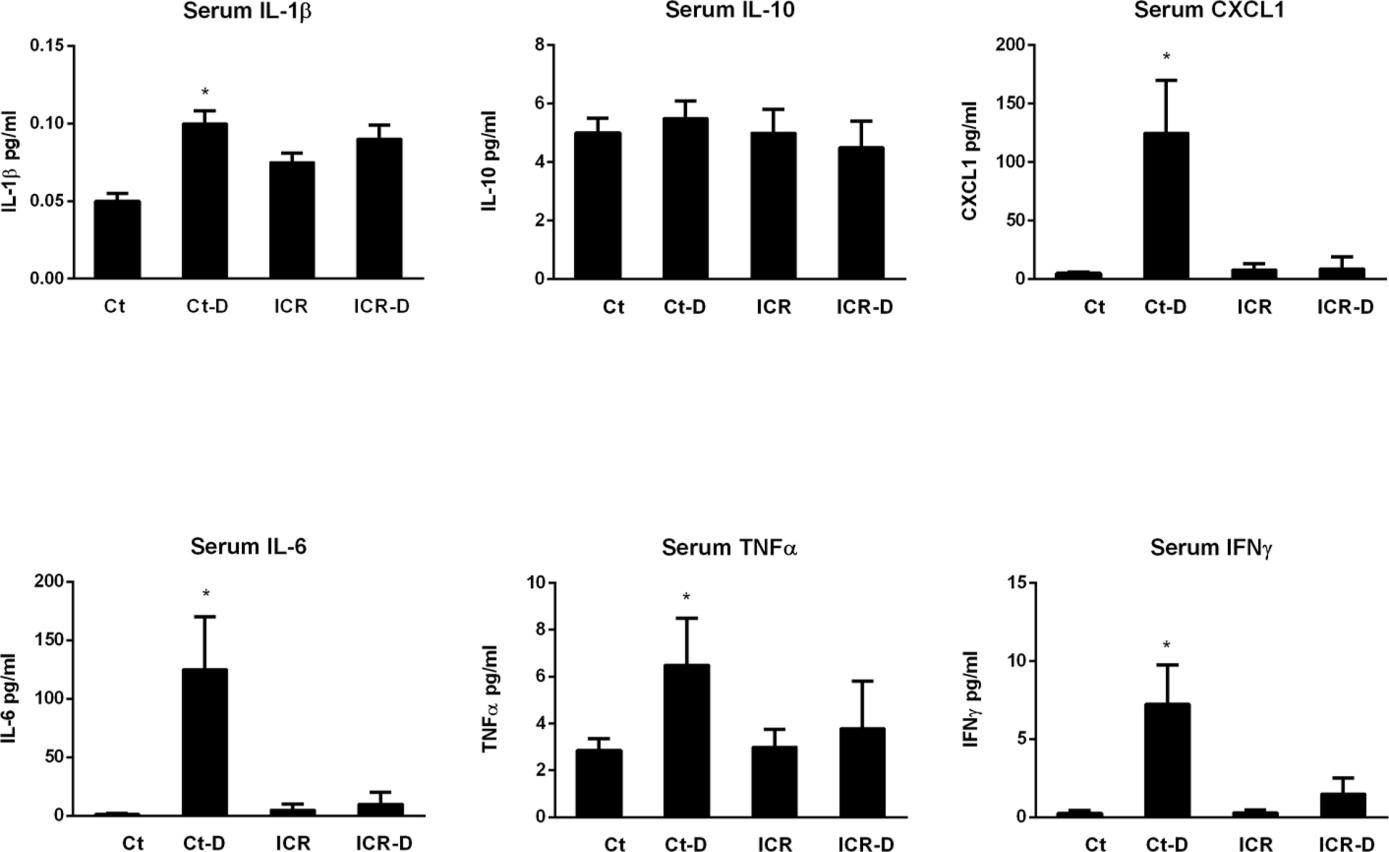
Serum cytokine levels. Ct-D groups had increased levels of IL-1β, CXCL1, IL-6, TNFα, and IFNγ compared with Ct, ICR, and ICR-D. Results are representative of 6 independent experiments. * p ≤ 0.05 relative to Ct, ICR, and ICR-D.

Serum levels of IL-1β, CXCL-1, IL-6, TNF-α, and IFN-γ were all elevated with DSS treatment in control mice (Fig 11). In contrast, the ICR-D group, despite having significantly worse colonic disease, showed no increases in serum cytokines. Serum IL-10 levels were similar across the four groups.

## Discussion

The purpose of this study was to evaluate immunologic changes in the gut following an ileocolic resection and to determine how the resected gut responds to an acute insult in the early post-operative period. The ileocolic resection performed in our animal model was designed to mimic the resection procedure commonly performed for patients with Crohn’s disease and, because intestinal healing is a dynamic process, both an early time point following surgery and a later time point were studied. In this study, we demonstrate that ileocolic resection leads to significant perturbations in immunologic function from the mucosal level to the draining lymphatics and systemically and that mice were more susceptible to intestinal insults following surgery.

Immune perturbations induced by ileocolic resection were studied at two time points following surgery to investigate both early and later immune responses. The typical immune response following an ileocolic resection is a dynamic process and is driven by the inflammatory insult of surgery and subsequent need for wound healing. Indeed, several differences were seen between the mice studied at 16 days post-op and those studied at day 31 post-op. Mice studied at 16 days post-op developed significant weight loss during the DSS cycle and this was associated with elevated levels of myeloperoxidase suggesting increased neutrophilic recruitment during the early healing phase. The time to occult blood positive stools following introduction of a dextran sodium sulfate insult was shorter in ICR groups than either controls or sham surgery control mice, suggesting that these findings were due to the bowel resection itself and not simply due to perioperative stress.

We hypothesized that surgery would lead to shifts in innate immune cell populations as part of the healing response and that these shifts might account for the severity of the post-surgery response to DSS. The reduction in colonic neutrophilic infiltration in the ICR mice in response to DSS, together with the changes in innate immune cell profiles and loss of colonic cytokine responses would suggest that immune cells recruited to the post-surgical gut are less inflammatory and also less responsive to acute insults than cells recruited in control mice. This lack of inflammatory responses by recruited cells could have resulted from the systemic immunosuppression that appears to occur as a consequence of surgery due to changes in various neuroendocrine, paracrine, and hormonal systems[12, 13] and contributed to the severity of the post-ICR DSS phenotype. The blunting of immunologic responses in the colon following ICR would be expected to have significant effects on the intestine’s ability to maintain homeostasis should a break in the gut barrier occur. Dextran sodium sulfate acts by breaking down the mucosal barrier and allowing bacterial influx into the lamina propria; thus any failure by the innate immune system to deal with this microbial influx would worsen colitis. Non-operative control animals responded to DSS with large increases in colonic pro-inflammatory cytokines [24]. However, following ICR, the colonic cytokine response to DSS in the surgical mice was near absent. This failure to respond with increased cytokine secretion in the ICR group could have been due to a decrease in bacterial invasion or alternatively to phenotypic shifts in the mononuclear phagocyte populations. In the steady state, macrophage and dendritic cell populations in the distal ileum and colon are relatively inflammation anergic [6]. However the lamina propria compartment of the gut is continuously replenished with circulating precursors allowing for plasticity in reactivity and function [9]. Previous studies have demonstrated that DSS treatment results in the recruitment of immune cells with an inflammatory phenotype that produce high amounts of TNFα [25]. Consistent with these findings, DSS treatment of non-operative controls in our study led to an increase in CD11b^+^ cells in the colonic lamina propria, a higher relative proportion of NOS2^+^F4/80^+^ cells, and increased tissue inflammatory cytokines. Although CD11b^+^ cells also increased in ICR mice, DSS challenge in ICR mice failed to evoke an inflammatory response suggesting that the newly recruited CD11c^+^CD11b^+^ immune cells may have been either non-responsive to microbial products or the local environment was one of immunosuppression.

IL-6 is a pleiotropic cytokine that has been associated with active inflammation. However, it has also been shown to be induced rapidly after injury in several different cell types, including intraepithelial lymphocytes, monocytes, and T cells, and contributing to epithelial repair in the gut [26]. Our findings of an increase in colonic levels of IL-6 in control and sham mice in response to DSS is similar to that reported by Kuhn et al [26]. Of interest however, is the finding that ICR mice did not show a similar increase in IL-6 in response to DSS, suggesting that immune responses to an acute insult are significantly perturbed following gut resection. Although these findings are intriguing, more studies are required to determine the underlying cellular mechanisms of the reduced immune responses seen in the ICR mice.

Regional and systemic immune responses to DSS colitis were also perturbed with ICR. Flow cytometry of mesenteric lymph node cells demonstrated changes in mononuclear populations with DSS treatment in non-operative controls and further, stimulation of these cells in culture with fecal slurry or LPS suggested that these cells were primed for inflammatory responses leading to secretion of IL-6, TNF-α, and IL-10. A primed inflammatory response to bacterial antigens with DSS treatment was expected as DSS acts to increase bacterial invasion into the lamina propria [27]. However, mice which had undergone ICR had altered MLN cellular populations compared with controls, and these cells showed a blunted IL-10 and TNFα response to stimulation with either LPS or a fecal slurry. However, in contrast to what was seen in colonic tissue, MLN from ICR mice demonstrated increased baseline levels of IL-6 and TNF-α compared with controls, and IL-6 secretion in response to LPS or the fecal slurry was enhanced in the ICR mice in the absence of DSS. Previous studies have shown that CD11b+ lamina propria mononuclear cells are a source of IL-6 [28], suggesting that the increase in this cell population in the MLN could have contributed to the enhanced levels of IL-6 seen in the ICR mice.

Data from patients with Crohn’s disease and other mouse models of gut resection show increased bacterial loads to occur in the neo-terminal ileum in conjunction with a recurrence of disease in that region ^5,18^. While it is believed that the loss of the ileocecal valve is the primary reason for this increased bacterial load, data from our study showing shifts in innate immune cells to also occur in this region suggests that localized altered immune–microbial interactions may also contribute to an increased bacterial load and possibly disease recurrence in this particular region. Further, decreased cytokine responses to microbial stimuli in MLNs and spleen of ICR mice when exposed to bacterial antigens also supports the concept of surgical-induced inappropriate responses to microbes.

In conclusion, results from this study show that mice undergoing an ileocolic resection are more susceptible to acute insults and further, that intestinal surgical resection results in the recruitment of innate immune cells into the colon that exhibit a non-responsiveness to microbial stimuli. In that innate immune dysfunction appears to be a major contributing factor to Crohn’s disease pathogenesis, understanding how intestinal resection modulates immune function and immune-microbial interactions may help explain why disease recurrence tends to occur at the localized anastomosis region.

## Abbreviations

CD: Crohn’s disease
Ct: control
DC: dendritic cells
DSS: dextran sodium sulfate
ICR: ileocolonic resection
LPS: lipopolysaccharide
MФ: macrophages
nTI: neo-terminal ileum
MPO: myloperoxidase
Treg: T regulatory cells
